# Supplementary material for: Deep learning identifies partially overlapping subnetworks in the human social brain
Source: Commun Biol. 2021 Jan 14;4:65. doi: 10.1038/s42003-020-01559-z (PMC7809473; doi:10.1038/s42003-020-01559-z)
Supplement: Supplementary file 1 — Supplementary Information [file 42003_2020_1559_MOESM1_ESM.pdf]

Supplementary Online Material

Deep learning identifies partially overlapping subnetworks in the human social brain

Hannah Kiesow, R. Nathan Spreng, Avram J. Holmes, M. Mallar Chakravarty
Andre F. Marquand, B.T. Thomas Yeo, Danilo Bzdok

	**Percent**	**Mean**	**SD**	**Range**
Age		55	7.5	40-70
Sex<br>Female<br>Male	 52.4<br>47.6			
Ethnic background<br>British<br>Irish<br>Other white background<br>Other	 91.7<br>2.7<br>2.3<br>3.3			
Age completed full time education		17	2.3	5-35
Body mass index (BMI)		26.7	4.3	16.1-63.6

Supplementary Table 1. Demographic information of the UK Biobank participants.

Social brain region	Abbreviation	MNI coordinates	Cluster
left anterior insula	AI_L	-34 19 0	intermediate
right anterior insula	AI_R	38 18 -3	intermediate
left amygdala	AM_L	-21 -4 -18	limbic
right amygdala	AM_R	23 -3 -18	limbic
anterior mid-cingulate cortex	aMCC	1 25 30	intermediate
left cerebellum	Cereb_L	-21 -66 -35	intermediate
right cerebellum	Cereb_R	28 -70 -30	intermediate
dorsomedial prefrontal cortex	dmPFC	-4 53 31	higher associative
left fusiform gyrus	FG_L	-42 -62 -16	visual sensory
right fusiform gyrus	FG_R	43 -57 -19	visual sensory
medial frontal pole	FP	1 58 10	higher associative
left hippocampus	HC_L	-24 -18 -17	limbic
right hippocampus	HC_R	25 -19 -15	limbic
left inferior frontal gyrus	IFG_L	-45 27 -3	intermediate
right inferior frontal gyrus	IFG_R	48 24 2	intermediate
left middle temporal gyrus	MTG_L	-56 -14 -13	higher associative
right middle temporal gyrus	MTG_R	56 -10 -17	higher associative
left middle temporal V5 area	MT/V5_L	-50 -66 5	visual sensory
right middle temporal V5 area	MT/V5_R	50 -66 6	visual sensory
left nucleus accumbens	NAC_L	-13 11 -8	limbic

Supplementary Table 2a. Social brain regions and their MNI coordinates. Social brain regions and their respective functional network as depicted in[1].

right nucleus accumbens	NAC_R	11 10 -7	limbic
posterior cingulate cortex	PCC	-1 -54 23	higher associative
posterior mid-cingulate cortex	pMCC	-3 -29 32	higher associative
precuneus	Prec	-1 -59 41	higher associative
left posterior superior temporal sulcus	pSTS_L	-56 -39 2	visual sensory
right posterior superior temporal sulcus	pSTS_R	54 -39 0	visual sensory
rostral anterior cingulate cortex	rACC	-3 41 4	limbic
left supplementary motor area	SMA_L	-41 6 45	intermediate
right supplementary motor area	SMA_R	48 6 35	intermediate
left supramarginal gyrus	SMG_L	-41 -41 42	intermediate
right supramarginal gyrus	SMG_R	54 -30 38	intermediate
left temporal pole	TP_L	-48 8 -36	higher associative
right temporal pole	TP_R	53 7 -26	higher associative
left temporo-parietal junction	TPJ_L	-49 -61 27	higher associative
right temporo-parietal junction	TPJ_R	54 -55 20	higher associative
ventromedial prefrontal cortex	vmPFC	2 45 -15	limbic

Supplementary Table 2b. Social brain regions and their MNI coordinates. Social brain regions and their respective functional network as depicted in[1].

Hyperparameter	Possible values
Bias	Yes, No
Epochs	15, 30
Learning rate	0.1, 0.01, 0.001, 0.0001
Optimizer	RMSprop, SGD, Adam, Adagrad

Supplementary Table 3. Considered hyperparameters before model training. A grid search of these hyperparameter values were considered for training the autoencoder architectures.

Hidden Subnetwork	Data split 1	Data split 2	Data split 3	Data split 4
1	0.892	0.878	0.872	0.873
2	0.794	0.782	0.805	0.898
3	0.805	0.810	0.802	0.745
4	0.845	0.872	0.886	0.830
5	0.824	0.816	0.790	0.759
6	0.865	0.843	0.868	0.859
7	0.913	0.922	0.897	0.860
8	0.646	0.729	0.692	0.773
9	0.885	0.885	0.839	0.812
10	0.810	0.780	0.779	0.778
11	0.816	0.825	0.840	0.825
12	0.826	0.797	0.755	0.709
13	0.643	0.653	0.632	0.745
14	0.695	0.701	0.672	0.698
15	0.821	0.846	0.846	0.847

Supplementary Table 4. Pearson's correlation coefficients computed between the estimated hidden subnetworks obtained from autoencoder algorithms applied to the discovery set from the original UK Biobank sample (n=~10,000) and several new replication data splits of 10,000 participants from the February/March UK Biobank release.

**Group**	**Social Trait**	**Data split 1**	**Data split 2**	**Data split 3**	**Data split 4**
less social female	Friendship satisfaction	0.396	0.356	0.411	0.294
	Living alone	0.208	0.547	0.608	0.255
	Loneliness	0.395	0.545	0.240	0.122
	Romantic partners	0.674	0.712	0.588	0.665
	Social job	0.488	0.480	0.748	0.479
	Social support	0.642	0.528	0.580	0.512
less social male	Friendship satisfaction	0.516	0.568	0.481	0.395
	Living alone	0.160	0.223	0.228	-0.006
	Loneliness	0.371	0.577	0.365	0.440
	Romantic partners	0.564	0.675	0.713	0.573
	Social job	0.542	0.429	0.551	0.433
	Social support	0.477	0.520	0.368	0.480
more social female	Friendship satisfaction	0.495	0.451	0.291	0.245
	Living alone	0.349	-0.042	-0.151	0.400
	Loneliness	0.242	0.383	0.324	0.231
	Romantic partners	0.486	0.497	0.547	0.262
	Social job	0.470	0.507	0.281	0.452
	Social support	0.323	0.372	0.282	0.446
more social male	Friendship satisfaction	0.240	0.232	0.156	0.101
	Living alone	0.517	0.351	0.299	0.238
	Loneliness	0.425	0.411	0.380	0.017
	Romantic partners	0.562	0.371	0.442	0.540
	Social job	0.570	0.543	0.432	0.463
	Social support	0.492	0.339	0.588	0.337

Supplementary Table 5. Pearson's correlation coefficients computed between predictive model estimates obtained from the discovery set of the original UK Biobank sample (n=~10,000) and those[2, 3] from several new replication data splits of 10,000 participants from the February/March UK Biobank release.

Social trait	less social females	more social females	less social males	more social males
Social support	0.295	0.289	0.298	0.265
Friendship satisfaction	0.306	0.245	0.285	0.309
Romantic partners	0.269	0.422	0.363	0.257
Loneliness	0.247	0.232	0.289	0.310
Living alone	0.293	0.270	0.282	0.227
Social job	0.301	0.273	0.305	0.251

Supplementary Table 6. Table of predictive model performances specific for each of the six social traits (with age covariate, see Sup. Table 7), computed in the original UK Biobank sample (n=~10,000).

Social trait	less social females	more social females	less social males	more social males
Social support	0.3041	0.2779	0.2777	0.2673
Friendship satisfaction	0.2820	0.2543	0.3027	0.2697
Romantic partners	0.2433	0.3030	0.2779	0.3079
Loneliness	0.2204	0.2193	0.3030	0.2707
Living alone	0.2406	0.2596	0.2727	0.2236
Social job	0.3078	0.2396	0.2997	0.2448

Supplementary Table 7. Table of predictive model performances specific for each of the six social traits (without age covariate), computed in the original UK Biobank sample (n=~10,000).

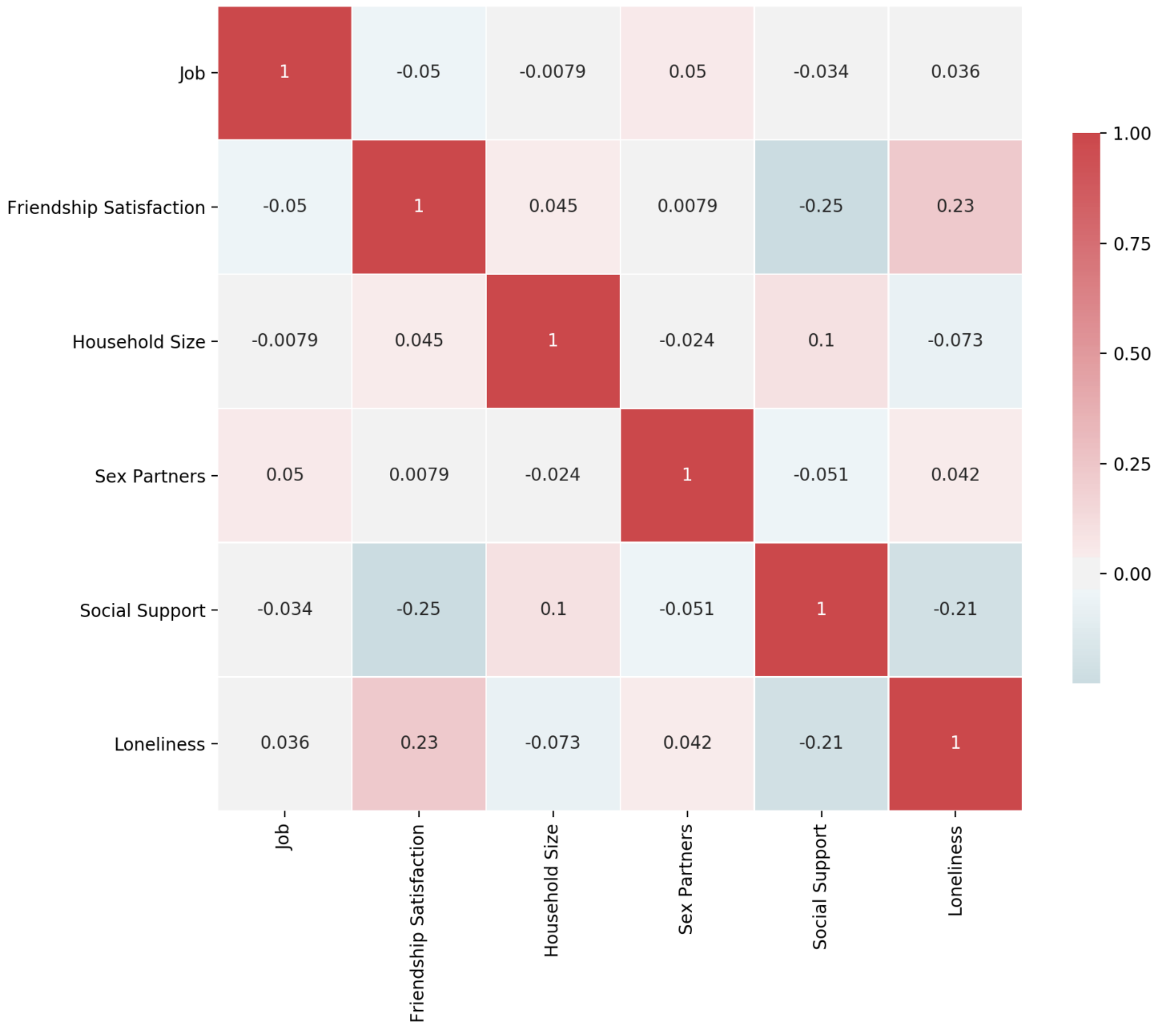

Supplementary Fig. 1: Correlation of social markers from the UK Biobank. Each square represents the correlation between a pair of examined social traits. Correlations are ordered by positive (red) and negative (blue) values. We computed 36 unique combinations of social traits in ~10,000 people, none of which exceed an absolute correlation strength of rho = 0.25.

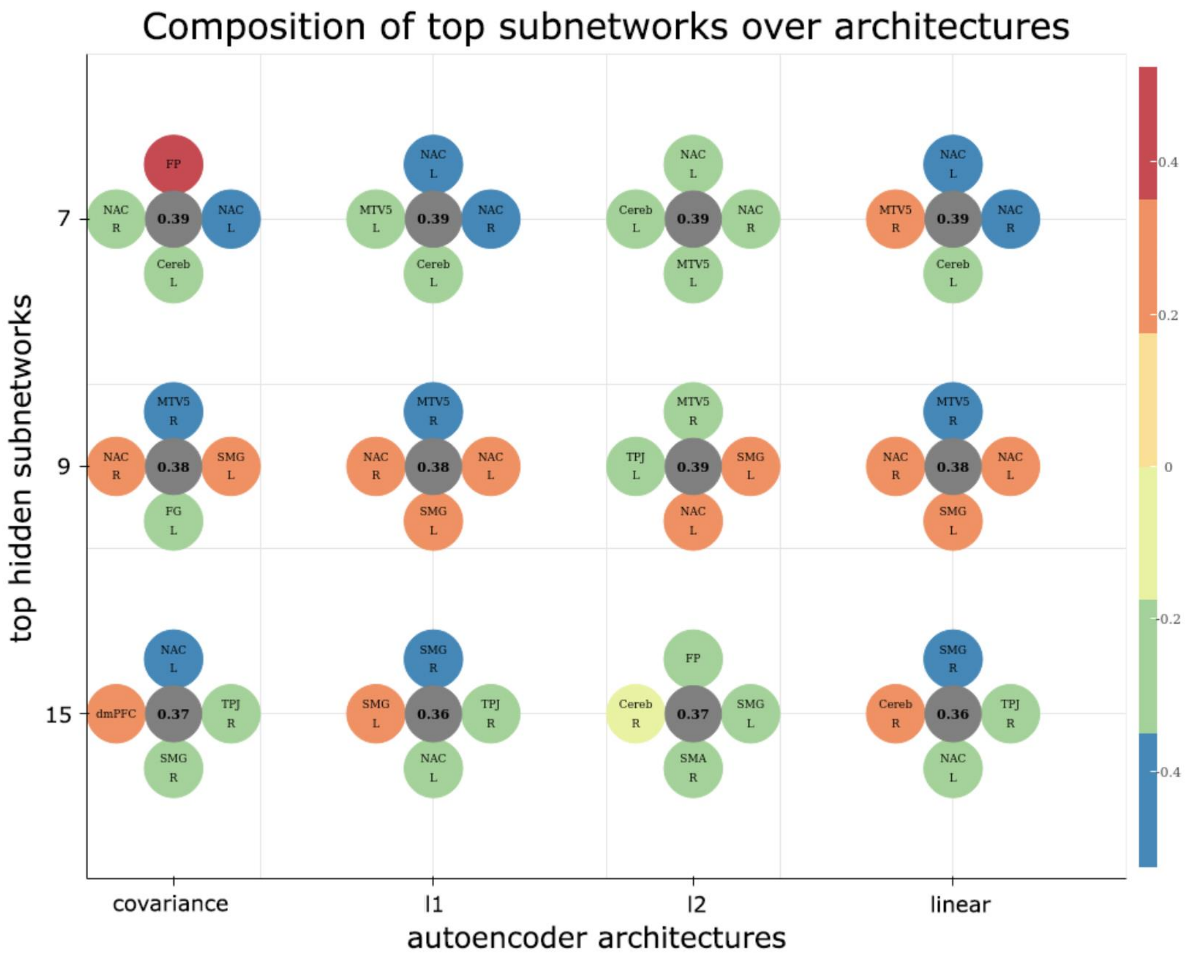

Supplementary Fig. 2. Distinct autoencoder architectures yielded similar region relevances in the top social subnetworks. Each of the social brain region relevances are depicted for the top hidden subnetworks. These relevances are stable, even when repeating the same analysis with the same data but using different autoencoder architectures. The four most relevant social brain regions are shown for the different autoencoder architectures (x-axis) and their three dominant subnetworks (y-axis). The values inside of the grey central circles show each subnetwork's importance (explained variance), while the four surrounding circles represent the strongest contributing brain regions for that specific hidden subnetwork. The colors of the highest contributing brain regions represent their contribution to the respective hidden subnetwork. At the level of specific regions composing the three top social subnetworks for a given model type, the linear, l1- and l2-penalized model architectures show nearly identical modeling solutions. Only the covariance model shows small differences, including higher region relevances for the pSTS and FG. These results show that the dominant three subnetworks are nearly identical for different autoencoder architectures, supporting the stability of the allocated region relevances of the prominent linear autoencoder.

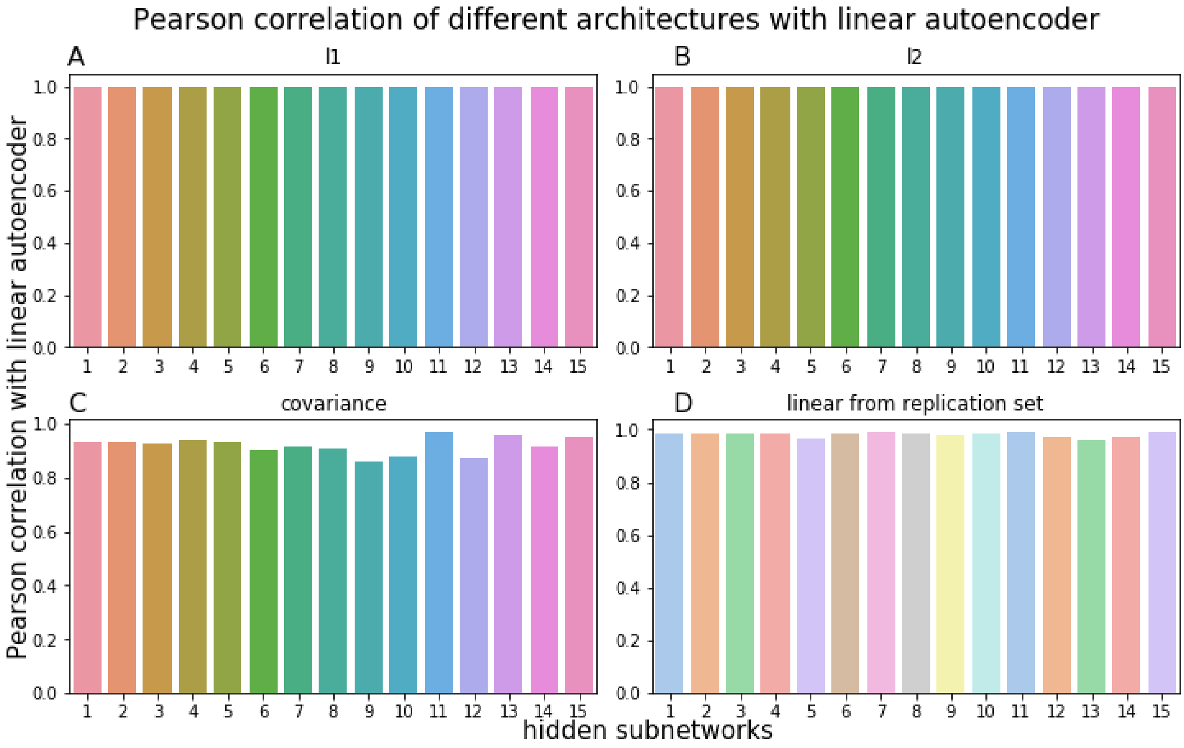

Supplementary Fig. 3: Stability of relevance allocated to the 36 regions for each particular subnetwork. The linear autoencoder architecture (cf. Fig. 4, column 4) is compared against three different competing model architectures (cf. Fig. 4, columns 1-3): (A) imposing additional parsimony by imposing exactly-zero region relevances, (B) imposing smaller absolute individual region relevances, and (C) imposing small correlation between subnetworks. In the plots, each bar displays the Pearson correlation between one subnetwork's set of 36 region relevances and that of the linear autoencoder's 36 region relevances. The results show that all subnetworks are highly correlated with the respective subnetworks from the linear autoencoder. Furthermore, the bottom right plot (D) shows the same correlation between the linear autoencoder trained and tested on independently drawn training and test sets. These high correlations show that our found subnetworks are highly robust between observed architectures (A-C) and datasets (D).

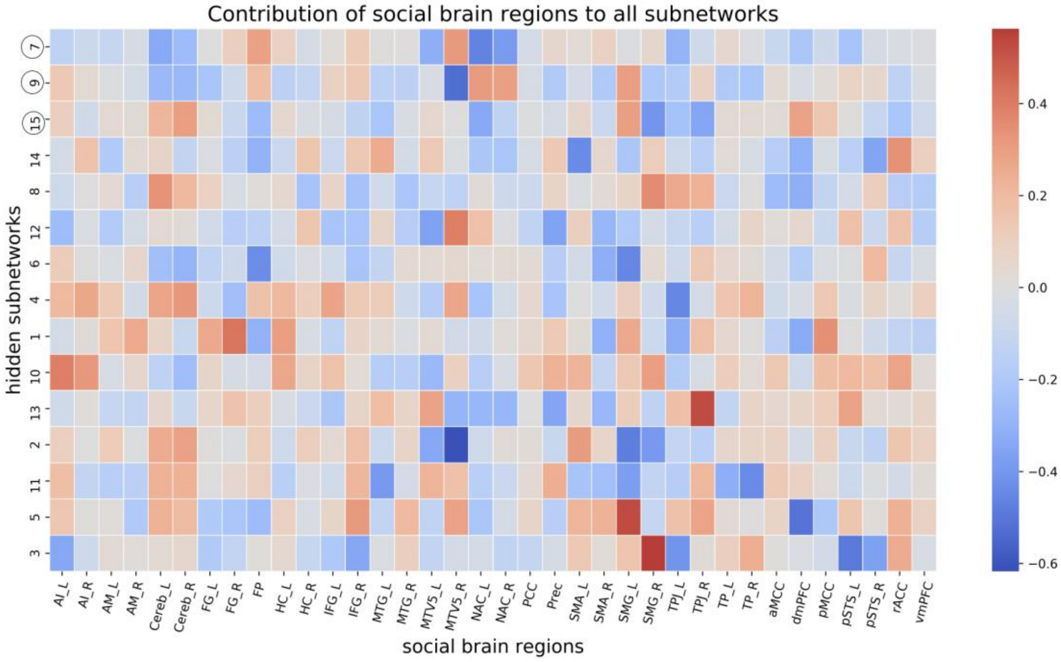

Supplementary Fig. 4: Relevance of each social brain region to all hidden social brain subnetwork representations. The heatmap depicts the different volume effects of each of the 36 social brain regions (x-axis) to all the learned hidden subnetworks (y-axis). The relevance of each particular brain region to the all hidden subnetworks is represented by their color for a respective combination of region and subnetwork. Blue colors depict less relevance to a specific subnetwork, while red colors depict more relevance to a given subnetwork.

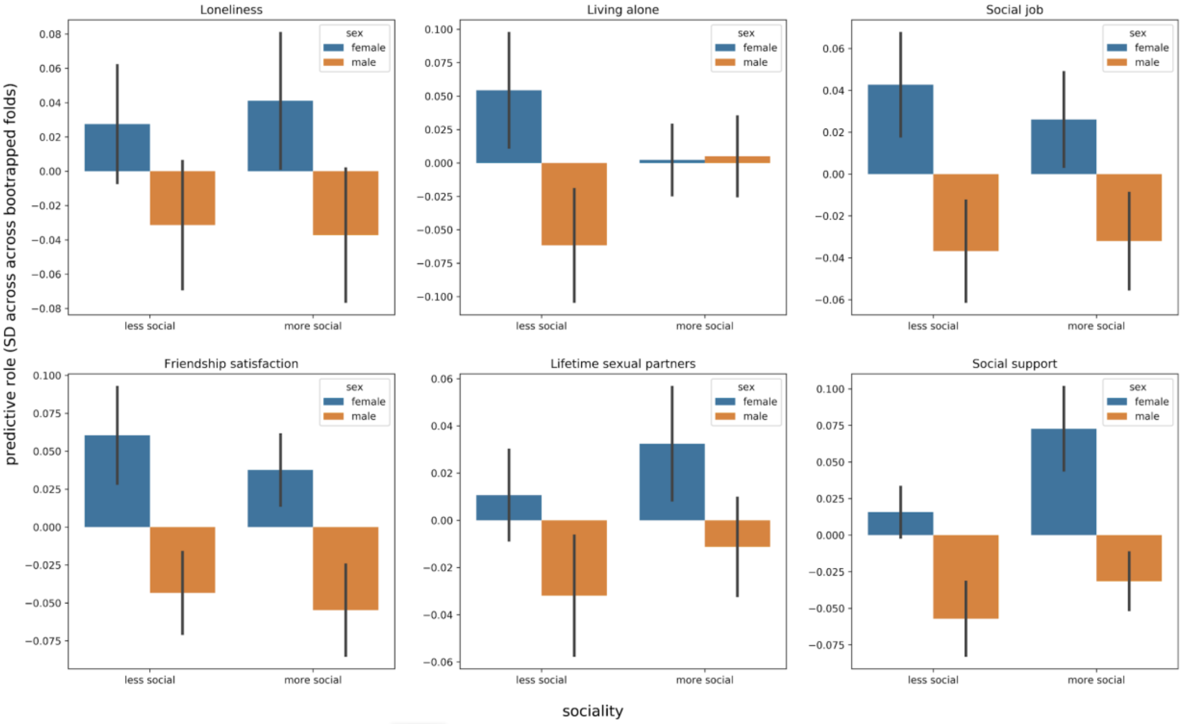

Supplementary Fig. 5: Predictive role of sex differences in subnetwork 10. Each plot depicts the predictive ability (y-axis) of subnetwork 10 for social and non-social individuals (x-axis), split by sex (males in blue and females in orange) for each social marker. Subnetwork 10 shows a sex difference especially for social support, friendship satisfaction and the lifetime number of sexual partners.

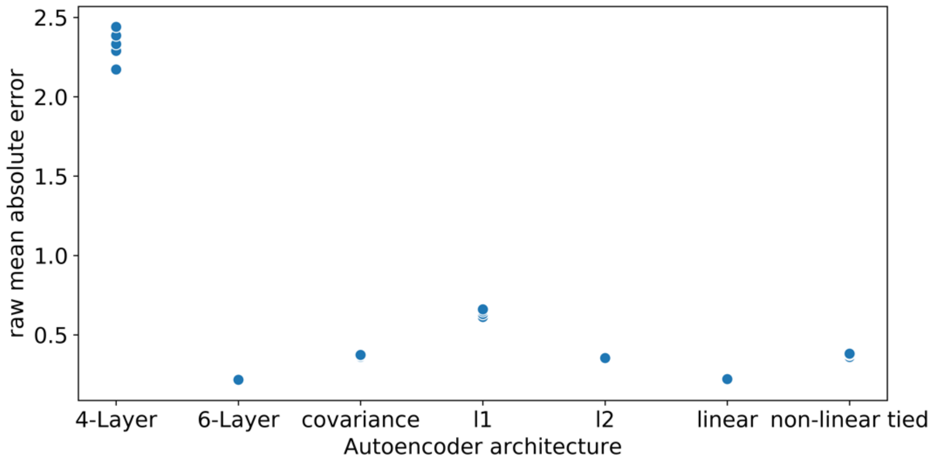

Supplementary Fig. 6: Model performance for different autoencoder neural networks from deep learning. After forming a given autoencoder model based on the training participants, all models' explained variance performances (i.e., mean absolute error) were evaluated on independent test participants. The explained variance of model performance across data splits are shown in the individual data points for each autoencoder architecture. The information provided here is identical to Figure 2, however displayed in a scatterplot format.

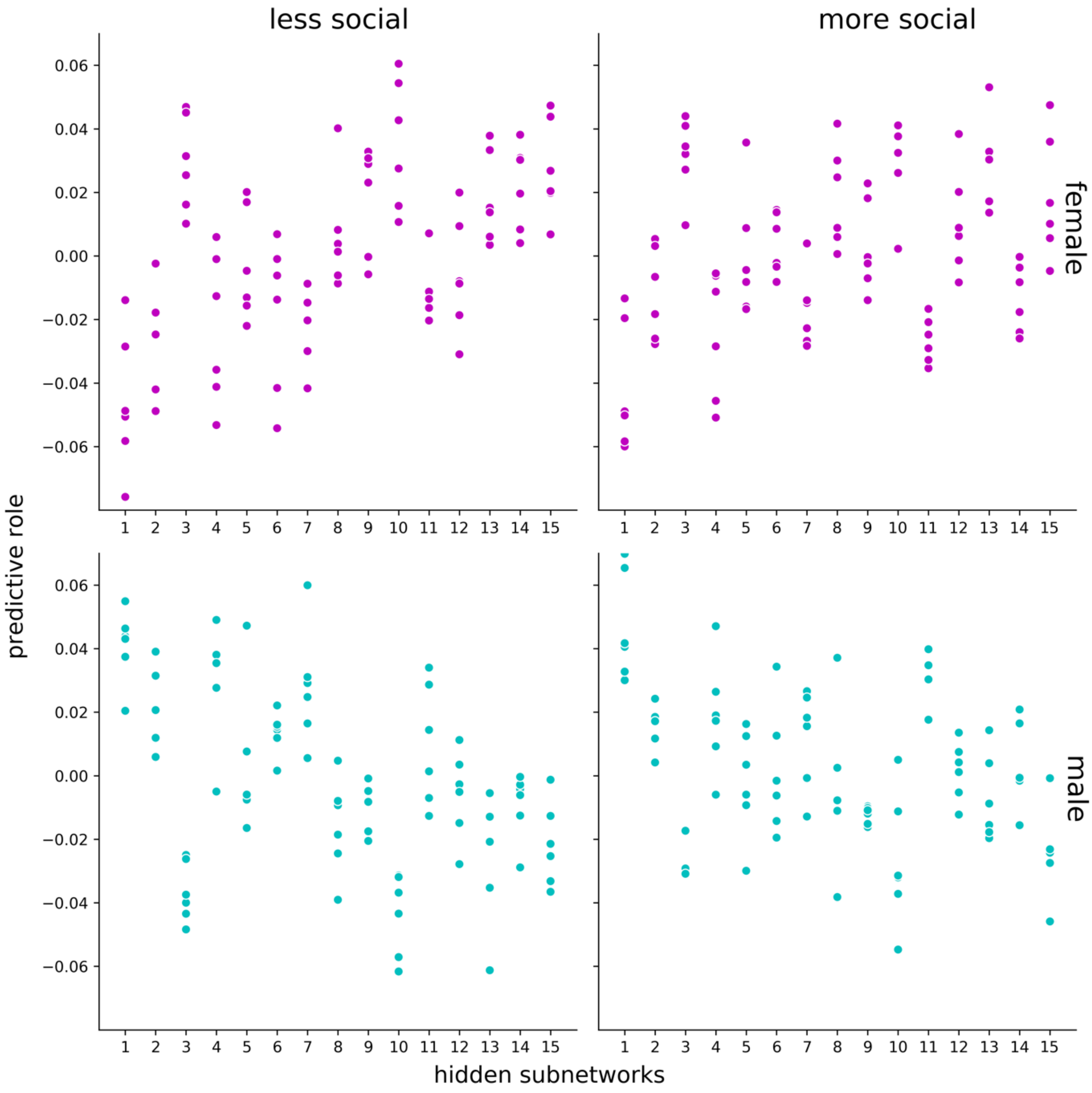

Supplementary Fig. 7: Overall predictive role of the hidden subnetworks for tracking more versus less social exchange. A logistic-loss classification algorithm was trained based on variation in subnetwork expressions across participants to learn predictive patterns for distinguishing the amount of regular social stimulation in men and women. The predictive contributions (*y axis*) corresponding to each subnetwork (*x axis*) are shown in the individual data points for degree of sociality for each of the four target groups. The information provided here is identical to Figure 6, however displayed in a scatterplot format.

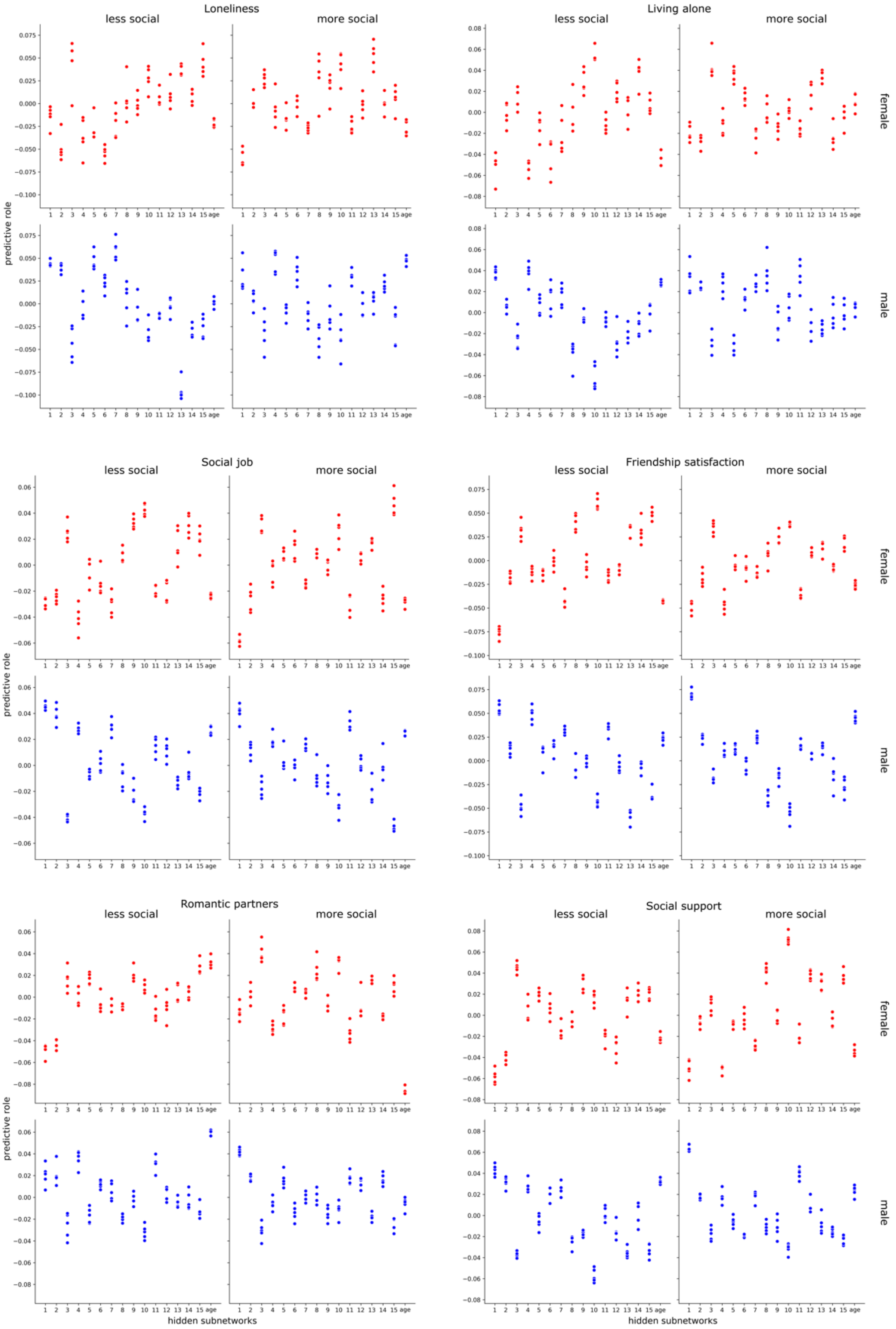

Supplementary Fig. 8: Specific predictive profile of the hidden subnetworks for tracking single social markers. The classification algorithm (cf. Fig. 6) was applied to learn predictive patterns separately for each social marker. The predictive contributions (*y axis*, units on z-scale) corresponding to each hidden subnetwork (*x axis*) are shown in the individual data points for each examined social trait, for each of the four target groups. The information provided here is identical to Figure 7, however displayed in a scatterplot format.

1. Alcala-Lopez D, *et al*. Computing the Social Brain Connectome Across Systems and States. *Cereb Cortex* **28**, 2207-2232 (2018).

2. Bzdok D. Classical Statistics and Statistical Learning in Imaging Neuroscience. *Front Neurosci* **11**, 543 (2017).

3. Hastie T, Tibshirani R, Friedman J. *The elements of statistical learning: data mining, inference, and prediction*. Springer Science & Business Media (2009).
