# Supplementary material for: Deep learning identifies partially overlapping subnetworks in the human social brain
Source: Commun Biol. 2021 Jan 14;4:65. doi: 10.1038/s42003-020-01559-z (PMC7809473; doi:10.1038/s42003-020-01559-z)
Supplement: Supplementary file 2 — Description of Additional Supplementary Files [file 42003_2020_1559_MOESM2_ESM.docx]

**Description of Additional Supplementary Files**

**File name:** Supplementary Data 1

**Description:** Within this source data file, we have created 7 different tabs, corresponding to the data that produced the figures. For Figure 3, however, we created two tabs labeled “3a” and “3b”, since the Figure contains two different data sources. Tab “3a” contains the source data for the left panel of Figure 3, and tab “3b” contains the source data for the right panel of Figure 3.
