# Supplementary material for: Deep learning identifies partially overlapping subnetworks in the human social brain
Source: Commun Biol. 2021 Jan 14;4:65. doi: 10.1038/s42003-020-01559-z (PMC7809473; doi:10.1038/s42003-020-01559-z)
Supplement: Supplementary file 4 — Reporting Summary [file 42003_2020_1559_MOESM4_ESM.pdf]

nature research

Corresponding author(s): Danilo Bzdok

Last updated by author(s): Oct 19, 2020

Reporting Summary

Nature Research wishes to improve the reproducibility of the work that we publish. This form provides structure for consistency and transparency in reporting. For further information on Nature Research policies, see Authors & Referees and the Editorial Policy Checklist .

Statistics

For all statistical analyses, confirm that the following items are present in the figure legend, table legend, main text, or Methods section.

n/a	Confirmed	
☐	☒	The exact sample size (n) for each experimental group/condition, given as a discrete number and unit of measurement
☐	☒	A statement on whether measurements were taken from distinct samples or whether the same sample was measured repeatedly
☐	☒	The statistical test(s) used AND whether they are one- or two-sided<br>*Only common tests should be described solely by name; describe more complex techniques in the Methods section.*
☐	☒	A description of all covariates tested
☐	☒	A description of any assumptions or corrections, such as tests of normality and adjustment for multiple comparisons
☐	☒	A full description of the statistical parameters including central tendency (e.g. means) or other basic estimates (e.g. regression coefficient) AND variation (e.g. standard deviation) or associated estimates of uncertainty (e.g. confidence intervals)
☐	☒	For null hypothesis testing, the test statistic (e.g. F, t, r) with confidence intervals, effect sizes, degrees of freedom and P value noted<br>*Give P values as exact values whenever suitable.*
☐	☒	For Bayesian analysis, information on the choice of priors and Markov chain Monte Carlo settings
☐	☒	For hierarchical and complex designs, identification of the appropriate level for tests and full reporting of outcomes
☐	☒	Estimates of effect sizes (e.g. Cohen's d, Pearson's r), indicating how they were calculated

Our web collection on statistics for biologists contains articles on many of the points above.

Software and code

Policy information about availability of computer code

Data collection	Data are provided as freely available resource available to all scientists. No software was used for data collection.
Data analysis	Software used for data analysis:<br>Keras (version: 2.4.0)<br>nilearn (version: 0.6.2)<br>sklearn (version: 0.21.3)<br>PySurfer (version: 0.10.0)<br>Seaborn (version: 0.11.0)<br>Bokeh (version: 1.3.4)<br>Brain Extraction Tool (part of FMRIB Software Library v6.0)<br>SIENAX (part of FMRIB Software Library v6.0)<br>MELODIC (part of FMRIB Software Library v6.0)<br>MCFLIRT (part of FMRIB Software Library v6.0)<br>ICA-based X-noiseifier (part of FMRIB Software Library v6.0)<br>FMRIB automatic segmentation tool (part of FMRIB Software Library v6.0)<br>FNIRT (part of FMRIB Software Library v6.0)<br>FAST (part of FMRIB Software Library v6.0)

For manuscripts utilizing custom algorithms or software that are central to the research but not yet described in published literature, software must be made available to editors/reviewers. We strongly encourage code deposition in a community repository (e.g. GitHub). See the Nature Research guidelines for submitting code & software for further information.

Data

Policy information about availability of data

All manuscripts must include a data availability statement. This statement should provide the following information, where applicable:

- Accession codes, unique identifiers, or web links for publicly available datasets
- A list of figures that have associated raw data
- A description of any restrictions on data availability

All used data are available to other investigators online (ukbiobank.ac.uk).

Field-specific reporting

Please select the one below that is the best fit for your research. If you are not sure, read the appropriate sections before making your selection.

☒ Life sciences ☐ Behavioural & social sciences ☐ Ecological, evolutionary & environmental sciences

For a reference copy of the document with all sections, see nature.com/documents/nr-reporting-summary-flat.pdf

Life sciences study design

All studies must disclose on these points even when the disclosure is negative.

Sample size	We used all subject data from the 10,000 UK Biobank release, no exclusions. No power calculation was needed in advance and we used all samples available (see below).
Data exclusions	We first identified the full set of ~10,000 participants who had also been imaged by UK Biobank, without exclusions. In the analyses on structural MRI data, we used all subjects who had that brain modality. In analyses on functional MRI data, we used all subjects who had that brain modality.
Replication	All cross-validated replications were carried out rigorously As such, we have performed rigorous cross-validation schemes: predictive patterns were identified in a larger split of training data and the build predictive models were then evaluated on an independent, unseen set of test participants in each fold of the cross-validation procedure.
Randomization	UK Biobank is an observational prospective epidemiological study, and the analyses in our study use all available subjects that fulfill the criteria described above. Hence there is no equivalent process of randomization that comes into this analysis (this is not a controlled randomised study).
Blinding	For exactly the same reasons (this is not a controlled randomised study), there is no step equivalent to blinding involved.

Reporting for specific materials, systems and methods

We require information from authors about some types of materials, experimental systems and methods used in many studies. Here, indicate whether each material, system or method listed is relevant to your study. If you are not sure if a list item applies to your research, read the appropriate section before selecting a response.

Materials & experimental systems

n/a	Involved in the study	
☒	☐	Antibodies
☒	☐	Eukaryotic cell lines
☒	☐	Palaeontology
☒	☐	Animals and other organisms
☐	☒	Human research participants
☒	☐	Clinical data

Methods

n/a	Involved in the study	
☒	☐	ChIP-seq
☒	☐	Flow cytometry
☐	☒	MRI-based neuroimaging

Human research participants

Policy information about studies involving human research participants

Population characteristics	10,000 UK biobank participants who are close to be representatively of the UK population, 47.5% men and 52.5% women, aged 40-69 years when recruited (mean age 54.9, standard deviation [SD] 7.5 years
Recruitment	Recruitment was done as part of the UKbiobank initiative by flyers and other means common in epidemiological research. For details on representativeness see Frey et al., 2017.
Ethics oversight	UK Biobank participants gave written, informed consent for the study, which was approved by the Research Ethics Committee under application 11/NW/0382.

Note that full information on the approval of the study protocol must also be provided in the manuscript.

Magnetic resonance imaging

Experimental design

Design type	Please see "Methods" for full details. Our analyses include data from Structural MRI (T1).
Design specifications	MRI data processing (to generate imaging-derived phenotypes) was done previously and is full described in references 4 (Miller).
Behavioral performance measures	Behavioral performance in the MRI scanner was not used in this study.

Acquisition

Imaging type(s)	Please see "Methods" for full details. Our analyses include data from Structural MRI (T1).
Field strength	3T
Sequence & imaging parameters	MRI data acquisition for the structural and functional modalities covers several pages of full detail, which is fully provided previously in reference 4 (Miller).
Area of acquisition	Siemens' auto-align was used to include the full brain in the imaged field-of-view; this was checked (and corrected if necessary) by the radiographer.

Diffusion MRI ☒ Used ☐ Not used

Parameters: Please see above for information about full details.

Preprocessing

Preprocessing software	See above (covered previously in full detail in Miller.
Normalization	See above (covered previously in full detail in Miller.
Normalization template	MNI 152
Noise and artifact removal	See above (covered previously in full detail in Miller.
Volume censoring	See above (covered previously in full detail in Miller. No volume censoring.

Statistical modeling & inference

Model type and settings	n/a
Effect(s) tested	n/a

Specify type of analysis: ☐ Whole brain ☒ ROI-based ☐ Both

Anatomical location(s): social brain atlas (Alcala-Lopez et al., 2018)

Statistic type for inference (See Eklund et al. 2016)	Inference was not carried out based on brain volumes parsed by the social brain atlas.
Correction	See above (Statistic type for inference).

Models & analysis

n/a	Involved in the study
☒	☐ Functional and/or effective connectivity
☒	☐ Graph analysis
☐	☒ Multivariate modeling or predictive analysis

Multivariate modeling and predictive analysis: We used logistic-loss predictive analysis for structural MRI data.
